# Enzymes Inhibition and Antidiabetic Effect of Isolated Constituents from *Dillenia indica*

**DOI:** 10.1155/2013/382063

**Published:** 2013-11-07

**Authors:** Sunil Kumar, Vipin Kumar, Om Prakash

**Affiliations:** ^1^Institute of Pharmaceutical Sciences, Kurukshetra University, Kurukshetra, Haryana 136119, India; ^2^Department of Pharmacy, School of Chemical Sciences and Pharmacy, Central University of Rajasthan, Ajmer 305801, India

## Abstract

*Aims*. This study was designed to investigate the enzyme inhibitory and antidiabetic activity for the constituents isolated from *Dillenia indica*. *Methods*. The leaves of *D. indica* were extracted with methanol and subjected to fractionation and chromatographic separation, which led to the isolation of seven compounds: betulinic acid (**1**), n-heptacosan-7-one (**2**), n-nonatriacontan-18-one (**3**), quercetin (**4**), **β** sitosterol (**5**), stigmasterol (**6**), and stigmasteryl palmitate (**7**). Among these isolates, compounds **1**, **4**, **5**, and **6** were evaluated for *in vitro* enzyme inhibition and compounds **4**, **5** and **6** were evaluated for antidiabetic activity in streptozotocin-nicotinamide induced diabetic mice. *Results*. Compounds **1**, **4**, **5**, and **6** showed 47.4, 55.2, 48.8, and 44.3% **α**-amylase inhibition, respectively, and 52.2, 78.2, 52.5, and 34.2% **α**-glucosidase inhibition, respectively, at the dose of 50 *µ*g/kg. Compounds **4**, **5** and **6** also showed significant (∗*P* < 0.05) antidiabetic activity in streptozotocin-nicotinamide induced diabetic mice at the dose of 10 mg/kg. *Conclusion.* These results provide evidence that *Dillenia indica* might be a potential source of antidiabetic agents.

## 1. Introduction 

*Diabetes mellitus* is a metabolic disorder resulting from a defect in insulin secretion, insulin action, or both. Worldwide, the total number of people with diabetes is projected to increase from 171 million in 2000 to 366 million in 2030 [1]. One therapeutic approach to decrease the hyperglycemia, especially after a meal, is to retard and reduce the digestion and absorption of ingested carbohydrates through the inhibition of carbohydrate hydrolyzing enzymes (**α**-amylase and *α*-glucosidase) in the digestive organs. As a result, these inhibitors could decrease the postprandial rise in blood glucose concentration [2]. *α*-Amylase is one of the enzymes in the digestive system that catalyses the breakdown of starch to maltose and finally to glucose, which is the only sugar that can be utilized by the body [3]. Therefore, natural inhibitors from the dietary plants show lower inhibitory effect against *α*-amylase activity and can be used as effective therapy for postprandial hyperglycemia with minimal side effects [4]. *α*-Glucosidase inhibitors can retard the liberation of glucose from dietary complex carbohydrates and delay glucose absorption, resulting in reduced postprandial plasma glucose levels and suppressed postprandial hyperglycemia [5]. So, *α*-amylase and *α*-glucosidase inhibitors are drug-design targets in the development of compounds for the treatment of diabetes, obesity, and hyperlipaemia [6]. Many plant extracts and phytoconstituents showed *in vitroα*-glucosidase inhibition effect [7]. Furthermore, herbal treatment is preferred for diabetes due to lesser side effects and low cost [8]. 

*Dillenia indica* (Family: Dilleniaceae) is commonly called “Elephant tree.” The bark and leaves have astringent effects. The fruit shows laxative properties and is used for relieving abdominal pain [9]. The juices of *D. indica* leaves, bark, and fruits are mixed and given orally for the treatment of cancer and diarrhea [10]. Fruits and leaves extracts of *D. indica *are reported to have antioxidant activity [11], CNS depressant activities [12], and anti-inflammatory activity [13] in mice. The alcoholic extract of the leaves of *D. indica* has antimicrobial activity [14]. Traditionally, the plant is also used for treatment of diabetes [15]. The methanolic extract also shows free radicals scavenging effect [16]. The methanolic leaves extract of plant shows antidiabetic activity in alloxan and streptozotocin induced diabetes [17, 18].

 As in our previous work, ethyl acetate fraction of methanolic leaves extract of plant also shows antidiabetic activity in streptozotocin and streptozotocin-nicotinamide induced diabetes [19]. So, phytoconstituents were isolated from the active fraction and their antidiabetic activity was checked *in vivo* as well as their enzyme inhibition effect against *α*-amylase and *α*-glucosidase.

## 2. Material and Methods

### 2.1. Plant Material

*Dillenia indica *leaves were collected from the campus of Kurukshetra University, Kurukshetra, India, and were identified by Dr. H. B. Singh, Scientist F & Head, Raw Material Herbarium & Museum, NISCAIR, New Delhi, India. A voucher specimen of the plant is preserved in the herbarium (NISCAIR/RHMD/Consult/-2009-10/1381/182/1).

### 2.2. Extract Preparation and Fractionation

**Figure 1:**
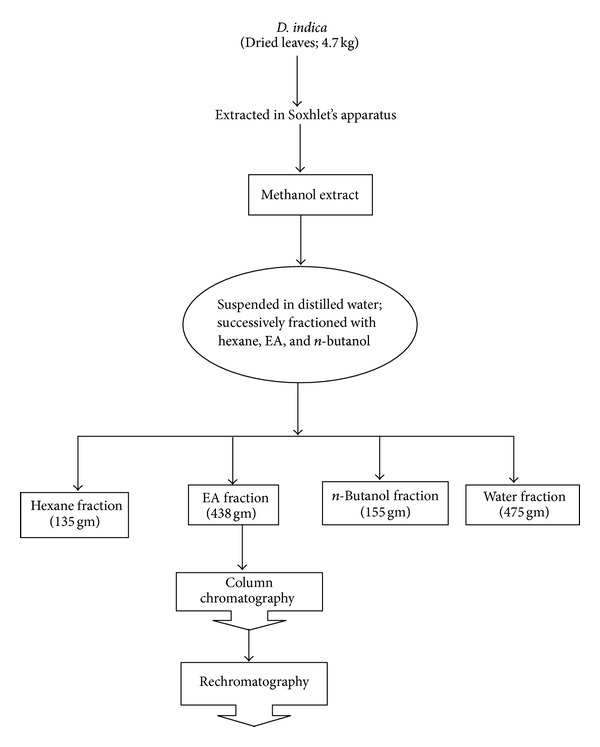
Schematic representation of extraction and fractionation of *D. indica. *

The collected leaves were washed with distilled water and dried in shade. The dried leave were powdered by using dry grinder and passed through sieve. The powered material (4.7 kg) was extracted with methanol by Soxhlet's apparatus. The extract was evaporated to dryness under reduced pressure at 45°C to give solid residues. The solid extract was suspended in water and successively extracted with hexane, ethyl acetate, and n-butanol by separating funnel (Figure 1). Each fraction was stored in airtight containers in refrigerator below 10°C for subsequent experiments.

### 2.3. Isolation of Phytoconstituents

The ethyl acetate fraction (50 g) was subjected to silica gel (number 60–120) column chromatography (CC) and eluted with pure hexane. Then it was further eluted successively, in order to increase polarity of the solvents in various combinations like hexane: chloroform; chloroform; chloroform: methanol. On the basis of TLC pattern, fractions DIC^23^ to DIC^29^ eluted with mobile phase (CHCl_3_ : CH_3_OH; 90 : 10) were mixed together, dried, and recrystallized to obtain 73 mg of whitish/colourless crystals of compound **1**. Compound **2** (71 mg of colourless amorphous powder) was obtained from fractions DIC^38^ to DIC^44^ after elution with mobile phase (CHCl_3_ : CH_3_OH; 75 : 25). Fractions (DIC^50^ to DIC^59^) eluted from the column with solvent system (CHCl_3_ : CH_3_OH; 60 : 40 & 50 : 50) gave 65 mg of pale yellow amorphous powder of compound **3**. Compound **4** (53 mg of pale yellow amorphous powder) was obtained from fractions DIC^73^ to DIC^80^ after elution with mobile phase (CHCl_3_ : CH_3_OH; 20 : 80). 

Fractions DIC^4^ to DIC^8^ were collected with mobile phase hexane: chloroform; 50 : 50 and fractions DIC^9^ to DIC^11^ were collected with mobile phase pure chloroform and were pooled on the basis of TLC. These were concentrated and dried in vacuum oven to obtain 210 mg of crude mass (DID) for rechromatography. Fractions (DID^11^ to DID^20^) eluted from the column with solvent system (chloroform : methanol; 98 : 2) gave 35 mg of whitish/colourless crystalline compound **5**. Fractions (DID^25^ to DID^31^) eluted from the column with solvent system (chloroform : methanol; 90 : 10) gave 41 mg of whitish/colourless crystalline compound **6**. 32 mg of colourless crystalline compound **7** was obtained from fractions (DID^40^ to DID^51^) after elution from the column with solvent system (chloroform : methanol; 75 : 25 and 70 : 30). 

### 2.4. Spectra of Isolated Phytoconstituents

#### 2.4.1. Betulinic Acid **(1)**

IR *γ*_max⁡_ (KBr): 3448, 2939, 2870, 2361, 1736, 1682, 1458, 1373, 1234, 1188, 1041, 887 cm^−1^.

^1^H NMR (CDCl_3_): *δ* 4.46 (1H, brs, C-29a) 4.48 (1H, brs, C-29b), 3.20 (1H, dd, *J* = 5.1 Hz, H-3*α*), 2.97 (1H, m, H-19), 1.60 (3H, s, Me-30), 1.28 (3H, brs, Me-23), 0.98 (3H, brs, Me-25), 0.95 (3H, brs, Me-26), 0.94 (3H, brs, Me-27), 0.73(3H, brs, Me-24).

+ve ESI MS *m/z*: 457 [M+1]^+^ (C_30_H_49_O_3_), 439 (M-H_2_O)^+^, 411 (M-COOH)^+^.

#### 2.4.2. n-Heptacosan-7-one **(2)**

IR *γ*_max⁡_ (KBr): 2916, 2847, 1705, 1466, 1373, 1324, 1112, 1049, 725 cm^−1^.

^1^H NMR (CDCl_3_): *δ* 2.34 (2 H, t, *J* = 7.5 Hz, H_2_-6), 2.01 (2H, t, *J* = 7.2 Hz, H_2_-8), 1.65 (10 H, brs, 5 × CH_2_), 1.55 (2H, m, CH_2_), 1.26 (32 H, brs, 16 × CH_2_),0.87 (3 H, t, *J* = 6.5 Hz, CH_3_-1), 0.81 (3 H, t, *J* = 6.3 Hz, CH_3_-27).

+ve ESI MS *m/z*: 395 [M+1]^+^ (C_27_H_55_O^−^). 

#### 2.4.3. n-Nonatriacontan-18-one **(3)**

IR *γ*_max⁡_ (KBr): 2924, 2854, 1706, 1458, 1288, 1250, 941, 725 cm^−1^.

^1^H NMR (CDCl_3_): *δ* 2.34 (2 H, t, *J* = 7.5 Hz, H_2_-17), 2.05 (2 H, t, *J* = 6.6 Hz, H_2_-19), 1.66 (4H, m, 2 × CH_2_), 1.32 (30 H, brs, 15 × CH_2_), 1.27 (34 H, brs, 17 × CH_2_), 0.89 (3H, t, *J* = 6.1 Hz, Me-1), 0.84 (3H, Me-39).

+ve ESI MS *m/z*: 563 [M+1]^+^, (C_39_H_79_O), 295.

#### 2.4.4. Quercetin **(4)**

IR *γ*_max⁡_ (KBr): 3394, 3310, 1659, 1605, 1558, 1520, 1450, 1381, 1311, 1257, 1196, 1095, 818, 795 cm^−1^.

^1^HNMR (DMSO): *δ* 7.81 (1H, brs, H-6′), 7.67 (1H, d, *J* = 2.0 Hz, H-2′), 6.9 (1H, d, *J* = 8.2 Hz, H-5′), 6.47 (1H, brs, H-8), 6.18 (1H, brs, H-6).

+ve ESI MS *m/z*: 303 [M+1]^+^, 302.

#### 2.4.5. *β*-Sitosterol **(5)**

IR *γ*_max⁡_ (KBr): 3441, 2942, 2362, 2340, 1694, 1542, 1460, 1385, 1032, 997, 785, 668 cm^−1^.

^1^HNMR (CDCl_3_): *δ* 5.34 (1H, d, *J* = 5.02 Hz, CH), *δ* 3.52 (1H, m), 1.00 (3H, s, CH_3_) *δ* 0.91 (3H, d, *J* = 6.4 Hz, CH_3_), 0.83 (3H, d, *J* = 6.4 Hz, CH_3_), 0.87 (3H, d, *J* = 6.4 Hz, Me), 0.89 (3H, d, *J* = 6.4 Hz), 0.81 (3H, d, *J* = 6.4 Hz, Me), *δ* 0.74 (3H, s, CH_3_).

+ve ESI MS *m/z*: 415 [M+1]^+^, 414 [M]^+^, 396, 255. 

#### 2.4.6. Stigmasterol **(6)**

IR *γ*_max⁡_ (KBr): 3412, 1665 cm^−1^.

^1^H NMR (CDCl_3_,): *δ* 5.36 (1H, m, CH), *δ* 5.15 (2H, m, CH=CH), *δ* 3.52 (1H, m, CH), 1.00 (3H, s, CH_3_) *δ* 0.93 (3H, d, *J* = 6 Hz CH_3_), 0.83 (3H, d, *J* = 5.4 Hz, CH_3_), 0.81 (3H, s, CH_3_), 0.79 (3H, d, *J* = 6.4 Hz, CH_3_), *δ* 0.67 (3H, s, Me).

+ve ESI MS *m/z*: 413 [M+1]^+^, 412 [M]^+^ (C_29_H_48_O), 394, 351, 271, 213, 173, 159, 145, 105, 91, 81, 55.

#### 2.4.7. Stigmasteryl Palmitate **(7)**

IR *γ*_max⁡_ (KBr): 2924, 2854, 2361, 1721, 1651, 1458, 1373, 1250, 1112, 1041, 964, 802, 725 cm^−1^.

^1^H NMR (CDCl_3_): *δ* 5.36 (1H, m, H-6) 5.20 (1H, m, H-22), 5.05 (1H, m, H-23), 3.54 (1H, brs, H-3*α*), 2.33 (2H, m, H_2_-21), 1.27 (26H, brs, 13 × CH_2_), 1.02 (3H, brs, Me-19), 0.95 (3H, d, *J* = 6.5 Hz, Me-21), 0.89 (3H, d, *J* = 6.5 Hz, Me-26), 0.87 (3H, d, *J* = 6.2 Hz, Me-27), 0.84 (3H, t, *J* = 6.6 Hz, Me-16), 0.82 (3H, d, *J* = 6.1 Hz, Me-29), 0.69 (3H, brs, Me-18).

+ve ESI MS *m/z*: 651 [M+1]^+^, 650 [M]^+^ (C_45_H_78_O_2_), 411, 397, 395, 381, 255.

### 2.5. *α*-Amylase Inhibition

Starch azure (2 mg) was suspended in a tube containing 0.2 mL of 0.5 M Tris-Hcl buffer (pH 6.9) containing 0.01 M calcium chloride (substrate). The tubes were boiled for 5 min and then preincubated at 37°C for 5 min. 1 mL of 0.1% of dimethyl sulfoxide was used to dissolve 1 mg of isolated fractions in order to obtain concentrations of 20, 40, 60, 80, and 100 *μ*g/mL. Then 0.2 mL of isolated fraction of a particular concentration was added in the tube containing the substrate solution. 0.1 mL of porcine pancreatic amylase in Tris-Hcl buffer (2 units/mL) was added to the tube containing the isolated fraction and substrate solution and all the processes were carried out at 37°C for 10 min. The reaction was stopped by adding 0.5 mL of 50% acetic acid in each tube. The reaction mixture was then centrifuged at 3000 rpm for 5 min and the absorbance of the resulting supernatant was measured at 595 nm spectrometrically [20]:

(1) The  α-amylase  inhibitory  activity  =(Ac+)−(Ac−)−(As−Ab)(Ac+)−(Ac−)×100,

where Ac^+^ is absorbance of 100% enzyme activity (only solvent with enzyme), Ac^−^ is absorbance of 0% enzyme activity (only solvent without enzyme), As is absorbance of test sample (with enzyme), and Ab is absorbance of blank (a test sample without enzyme), respectively. 

### 2.6. *α*-Glucosidase Inhibition

*α*-Glucosidase (50 *μ*L, 0.5 U/mL) and 0.2 M potassium phosphate buffer (pH 6.8, 50 *μ*L) were mixed with test sample (at various concentrations). After incubation at 37°C for 15 min, 3 mM pNPG (100 *μ*L) was added. The reaction was incubated again at 37° for 10 min and then stopped by 0.1 M Na_2_CO_3_. The absorption of 4-nitrophenol was measured at 405 nm [21]. The reaction mixture without sample was used as a control, and the mixture without substrate was used as a blank. 

The percentage inhibition of *α*-glucosidase was calculated as follows:

 Inhibition  rate (%) = {1 − ((Abs  sample − Abs  blank)/(Abs  control))} × 100,

where Abs sample is the absorbance of the experimental sample, Abs blank is the absorbance of the blank, and Abs control is the absorbance of the control.

### 2.7. Antidiabetic Activity

Noninsulin dependent diabetes (NIDDM) was induced in overnight fasted mice by a single intraperitoneal injection of 100 mg/kg STZ, 15 min after the i.p. administration of 240 mg/kg nicotinamide. Hyperglycemia was confirmed by the elevated blood glucose levels determined at 72 h and then on day 7 of the injection. Only mice confirmed with permanent NIDDM were used in the antidiabetic study [22]. For screening antidiabetic effect of isolated phytoconstituents, the overnight fasted mice were divided into six groups with five animals each and treated orally once as follows:

 group I: normal healthy control: received only vehicle (Tween 80, 2% v/v);
 group II: diabetic control: received only vehicle (Tween 80, 2% v/v);
 group III: diabetic mice received quercetin (10 mg/kg body wt.);
 group IV: diabetic mice received *β*-sitosterol (10 mg/kg body wt.);
 group V: diabetic mice received stigmasterol (10 mg/kg body wt.);
 group VI: diabetic mice received glibenclamide (10 mg/kg).

Experiments were performed in mice that had been fasted overnight (deprived of food for at least 12 h but allowed free access to water). Single dose of drug solutions or vehicle was administered orally by gastric intubation. The effect of vehicle, compounds, and standard drug on blood glucose level was determined in the animals at 0, 4, 8, and 24 h after daily oral administration. Blood samples were taken from the tip of the tail of the mice of different groups under mild ether anesthesia and glucose levels were determined by using blood glucose test strips with elegance glucometer (Frankenberg, Germany).

### 2.8. Statistical Analysis

All the results are presented as mean ± standard error of mean (SEM). The statistical analysis involving two groups was evaluated by means of Student's *t*-test, whereas one-way analysis of variance (ANOVA) followed by Dunnett's multiple comparison posttest was used for statistical comparison between control and various treated groups. Statistical significance was accepted at the *P* < 0.05 values.

## 3. Results and Discussion

**Figure 2:**
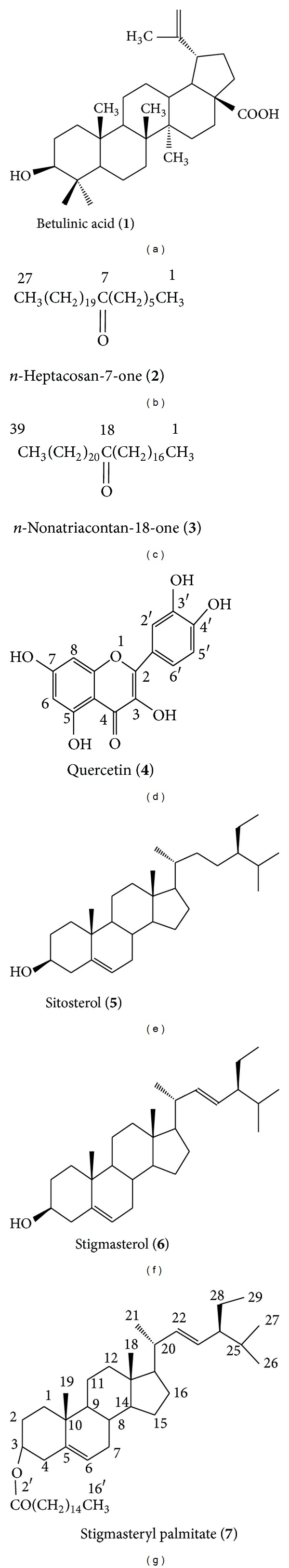
Chemical structures of isolated phytoconstituents.

The chemical structures of isolated constituents were established after interpretation of spectral data. Their melting points were measured and thin layer chromatography study was done as shown in Table 1. From aggregating all data, the chemical structures of seven compounds, betulinic acid, n-heptacosan-7-one, n-nonatriacontan-18-one, quercetin, *β*-sitosterol, stigmasterol, and stigmasteryl palmitate, were established as shown in Figure 2. 

In the present study, *in vitro α*-amylase and *α*-glucosidase inhibition effect of betulinic acid, quercetin, *β*-sitosterol, and stigmasterol was studied at 50 *μ*g/mL concentration. The phytoconstituents showed good *in vitro* enzyme inhibition activity. Betulinic acid, quercetin, *β*-sitosterol, and stigmasterol showed 47.4  ±  4.3, 55.2  ±  4.4, 48.8  ±  2.3, and 44.3  ±  2.4% *α*-amylase inhibition, respectively, and 52.2  ±  3.8, 78.2  ±  2.6, 52.5  ±  2.4, and 34.2  ±  3.79% *α*-glucosidase inhibition, respectively, at the dose of 50 *μ*g/kg (Table 2). Triterpenoids, flavonoids, and steroids isolated from various plants have also shown *α*-glucosidase inhibition activity [7]. *α*-Glucosidase is an exo-type carbohydrase distributed widely in microorganisms, plants, and animal tissues [23], which catalyzes the liberation of *α*-glucose from the nonreducing end of the substrate. Inhibiting this enzyme slows the elevation of blood sugar following a carbohydrate meal [5]. It is a membrane bound enzyme present in the epithelium of the small intestine, which works to facilitate the absorption of glucose by the small intestine by catalyzing the hydrolytic cleavage of oligosaccharides into absorbable monosaccharides. By inhibition of *α*-glucosidase in the intestine, the rate of hydrolytic cleavage of oligosaccharide is decreased and the process of carbohydrate digestion spreads to the lower part of small intestine [7]. This spreading of digestion process delays the overall absorption rate of glucose into the blood.

Streptozotocin possesses diabetogenic properties mediated by pancreatic beta cell destruction and this compound has been widely used to induce diabetes in experimental animals [24]. The isolated phytoconstituents quercetin, *β*-sitosterol, and stigmasterol from *D. indica* were also evaluated for type-2 diabetic in mice. The effect of compounds and glibenclamide on blood glucose level was determined in the animals at 0, 4, 8, 24 h after daily oral administration at the dose of 10 mg/kg. Quercetin, *β*-sitosterol, and stigmasterol showed significant reduction in blood glucose level as compared to the control group (Table 3). 

In our previous works, methanolic leaves extract *D. indica* had shown antidiabetic and antihyperlipidemic effect in alloxan and streptozotocin induced diabetic rats [17, 18]. Histopathological analysis showed that the extract has protective effect on vital organs (liver, kidney, and pancreas) in alloxan induced diabetic rats [17]. Further, fractionation of methanolic leaves extract was done as shown in Figure 2 and ethyl acetate fraction was evaluated for antidiabetic and hypolipidemic activities. The ethyl acetate fraction showed significant antidiabetic and hypolipidemic effect at a dose of 400 mg/kg in both type 1 and type 2 diabetic rats [19]. For confirmation of phytoconstituents responsible for activity of the plant, the ethyl acetate fraction of methanolic leaves extract was subjected to column chromatography and seven compounds were isolated. Betulinic acid, quercetin, *β*-sitosterol, and stigmasterol showed significant (*P* < 0.05) *α*-amylase and *α*-glucosidase inhibition. Quercetin, *β*-sitosterol, and stigmasterol also showed significant (*P* < 0.05) antidiabetic effect in streptozotocin-nicotinamide induced diabetic mice. It is concluded that betulinic acid, quercetin, *β*-sitosterol, and stigmasterol are responsible for antidiabetic property of *D. indica*. So, the results of present study confirmed that *D. indica* is a potential antidiabetic plant and it should be added in herbal formulations for management of diabetes.

## Figures and Tables

**Table 1:** Phytoconstituents isolated by column chromatography.

Sr. number	Compounds	m. p. (°C)	TLC solvent system	*R*_*f*_ value
1	Betulinic acid	297–300	Chloroform : methanol : formic acid (98 : 2 : 2)	0.35
2	n-Heptacosan-7-one	88–90	CHCl_3_ : MeOH (95 : 5)	0.53
3	n-Nonatriacontan-18-one	89–92	CHCl_3_ : MeOH (95 : 5)	0.42
4	Quercetin	310–313	CHCl_3_ : MeOH (95 : 5)	0.44
5	*β*-Sitosterol	137–139	CHCl_3_ : MeOH (90 : 10)	0.55
6	Stigmasterol	164–167	CHCl_3_ : CH_3_OH (90 : 10)	0.45
7	Stigmasteryl palmitate	94–97	Chloroform : methanol (95 : 5)	0.64

m.p.: melting point, *R*_*f*_: retention factor.

**Table 2:** Enzyme inhibition activity of isolated constituents.

Constituents	Conc.	*α*-Amylase (% inhibition)	*α*-Glucosidase (% inhibition)
Betulinic acid	50 *µ*g/mL	47.4 ± 4.3*	52.2 ± 3.8*
Quercetin	50 *µ*g/mL	55.2 ± 4.4*	78.2 ± 2.6*
*β*-Sitosterol	50 *µ*g/mL	48.8 ± 2.3*	52.5 ± 2.4*
Stigmasterol	50 *µ*g/mL	44.3 ± 2.4*	34.2 ± 3.7

Data represent means ± SEM, *P < 0.05.

**Table 3:** Antidiabetic effect of isolated phytoconstituents in STZ-NIC induced diabetic mice.

Groups/treatment	Blood glucose level (mg/dL)			
	0 h	4 h	8 h	24 h
(I) Vehicle	85.5 ± 3.2	85.94 ± 2.73	86.2 ± 2.4	85.26 ± 1.77
(II) STZ-NIC	237.23 ± 3.83	239.24 ± 4.32	239.83 ± 5.86	241.87 ± 4.44^a^
(III) STZ-NIC + quercetin	204.34 ± 5.56	173.5 ± 5.25*	162.23 ± 3.57*	165.53 ± 5.57*
(IV) STZ-NIC + *β*-sitosterol	225.68 ± 3.78	142.34 ± 3.4*	139.5 ± 3.7*	140.27 ± 3.57*
(V) STZ-NIC + stigmasterol	207.93 ± 2.85	158.69 ± 3.84	147.25 ± 4.31*	148.38 ± 3.72*
(VI) STZ-NIC + std.	224.37 ± 4.37	151.37 ± 2.43*	128.47 ± 3.72*	121.25 ± 5.1*

Data represent means ± SEM. *P < 0.05 when groups (III) to (VI) are compared with diabetic control, that is, group (II). Std.: standard drug (glibenclamide). ^a^ *P* < 0.05, when group (II) is compared with group (I).
